# Neck Dissection Timing in Transoral Robotic or Laser Microsurgery in Oropharyngeal Cancer: A Systematic Review

**DOI:** 10.1177/2473974X221131513

**Published:** 2022-10-11

**Authors:** Jai Parkash Ramchandani, Aina Brunet, Nikoleta Skalidi, Jack Faulkner, Aleix Rovira, Ricard Simo, Jean-Pierre Jeannon, Asit Arora

**Affiliations:** 1 King’s College London, London, England; 2 Department of Otorhinolaryngology and Head and Neck Surgery, Guy’s and St Thomas NHS Foundation Trust, London, England

**Keywords:** transoral robotic surgery, transoral laser microsurgery, neck dissection, fistula, hemorrhage, oropharyngeal cancer

## Abstract

**Objective:**

This review assesses the effect on intra- and postoperative patient outcomes of the timing of neck dissection in relation to transoral surgery. Outcome measures include postoperative bleeding, intra- and postoperative fistula formation, and disease-specific and overall survival.

**Data Sources:**

A search was conducted across the MEDLINE, Embase, US National Library of Medicine, and Cochrane databases with search terms in July 2021.

**Review Methods:**

Articles that conformed with specified inclusion criteria were included. Included articles were scanned for bias with the ROBINS-I tool.

**Results:**

Nineteen articles were selected for qualitative analysis, including 546 patients who had neck dissection in conjunction with transoral robotic surgery/transoral laser microsurgery (TORS/TLM). Seventy-one (18%) patients had neck dissection prior to TORS/TLM, 39 (10%) had neck dissection performed after TORS/TLM, and 281 (72%) had concurrent procedures. In patients with neck dissection before TORS/TLM, 3% experienced major postoperative bleeding, and fistula rates were 0%. In the cohort with neck dissection after TORS/TLM, 3% experienced minor postoperative hemorrhage, and 8% had intraoperative fistulae. In the concurrent cohort of patients, 1% had major postoperative bleeds and 0.3% had minor bleeds, while 4% developed intraoperative fistulas and 0.3% developed postoperative fistulas.

**Conclusion:**

Current evidence indicated that there appears to be no correlation between timing of neck dissection and complications. This systematic review found insufficient data to comment on whether the timing of neck dissection in relation to TORS/TLM affects the outcomes of patients.

Since the 1990s, the incidence of head and neck cancer has increased by 33% in the United Kingdom.^1^ Oropharyngeal squamous cell carcinoma (OPSCC) is a significant contributor to this dramatic rise,^2^ and it is thought that the increasing prevalence of OPSCC is driven by human papilloma virus.^3^ In addition, cervical lymph node metastasis is a common clinical finding at presentation. Current evidence indicates that between 50% and 70% of patients presenting with OPSCC will have lymph node metastasis in the neck.^4^

In the last few decades, there has been a shift of treatment paradigm from nonsurgical treatment to transoral surgical resection in patients with human papilloma virus–associated OPSCC. Improved outcomes of transoral robotic surgery (TORS)/transoral laser microsurgery (TLM) procedures and achieving primary resection with minimal morbidity are factors driving the increased popularity of these procedures.^5^ However, the timing of neck dissection (ND) in conjunction with these primary resection modalities remains controversial. Currently, there are no universally accepted guidelines or consensus for ND timing in patients undergoing TORS for OPSCC.^6^

ND has been performed concurrently, before, or after the primary tumor resection. Each technique is thought to have its own advantages and drawbacks (**Table 1**). Performing concurrent TORS/TLM and ND allows for single-session treatment. This will reduce the patient’s anaesthetic risk, overall hospital stay, and associated costs and may reduce the risk of delay of adjuvant therapy.^7^ Performing ND before primary resection allows vessel ligation before the TORS/TLM procedure, which may reduce hemorrhage intra- and postoperatively.^8^ It has been hypothesized that performing ND after TORS/TLM resection reduces fistula formation.^9^ Moreover, it provides an opportunity to address any close or positive resected margins in the histopathology report.^8^

**Table 1.** Advantages of Performing Neck Dissection Before, Concurrently, or After Transoral Surgery.

Before	Concurrent	After
Ligation of vessels to reduce hemorrhage during resection	Single theater session	Address close/positive margins following initial resection
	Reduced patient anesthetic risk	
	Reduced costs of surgery	
	No delay to adjuvant therapy	

The purpose of this review is to assess the impact of the timing of ND in relation to oropharyngeal cancer TORS/TLM on intra- and postoperative complications. These complications include postoperative bleeding, intra- and postoperative fistula formation, disease-specific survival (DSS), overall survival (OS), and recurrence rates.

## Methods

The systematic review is reported in accordance with the PRISMA guidelines (Preferred Reporting Items for Systematic Reviews and Meta-analyses) via methodology described in the *Cochrane Handbook for Systematic Reviews of Interventions*. A protocol was developed and peer reviewed locally before being registered on the PROSPERO database (CRD42021233780).

### Search Strategy

**Figure 1.**
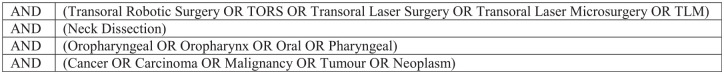
A list of search terms used for this review.

A search was conducted across the MEDLINE, Embase, US National Library of Medicine, and Cochrane databases with the search terms indicated (**Figure 1**), from their inception to July 2021 when the search was performed. The references of included articles were also searched.

### Study Selection

The articles filtered by the search strategy were considered in conformance with the following inclusion criteria:

- Primary studies
- Written in the English language (or provided English translations)
- Patients treated for a primary oropharyngeal cancer
- Patients undergoing TORS/TLM for primary resection in conjunction with an ND
- ND performed conventionally and not as robot-assisted procedures
- Timing of the ND specified as concurrent, before, or after TORS/TLM
- Results include surgical complications and functional patient-related outcomes

Studies describing TORS/TLM and ND in the salvage setting were excluded, and case reports were included. The main outcome measures were rates of postoperative hemorrhage, intra- and postoperative fistula formation, DSS, OS, and recurrence.

### Study Evaluation

Two reviewers (J.P.R. and A.B.-G.) were involved in the study selection process to ensure that no articles were missed. Any disagreement was resolved by discussion. Data from all the included articles were scanned independently by J.P.R. and A.B.-G. for bias per the ROBINS-I tool,^10^ and disagreement was resolved by discussion. ROBINS-I tool assesses bias within articles according to 7 domains:

- Bias due to confounding
- Bias in selection of participants into the study
- Bias in classification of intervention
- Bias due to deviations from intended interventions
- Bias due to missing data
- Bias in measurement of outcomes
- Bias in selection of results

This was in accordance with guidance from the *Cochrane Handbook*.^11^

### Categorization of ND

**Figure 2.**
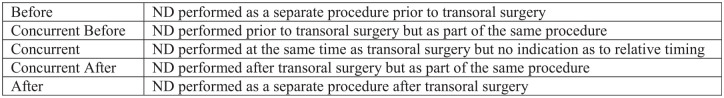
Definitions of categories in which patients were assigned. ND, neck dissection.

Timing of ND in relation to TORS/TLM was divided into 5 categories: before, concurrent before, concurrent, concurrent after, and after (**Figure 2**). In articles with patients who had concurrent procedures, it was not indicated whether the ND was performed before or after the TORS/TLM procedure, and so patients were grouped into a unified “concurrent” category. The Clavien-Dindo classification was used to assess complications among patients in the different cohorts.

## Results

**Figure 3.**
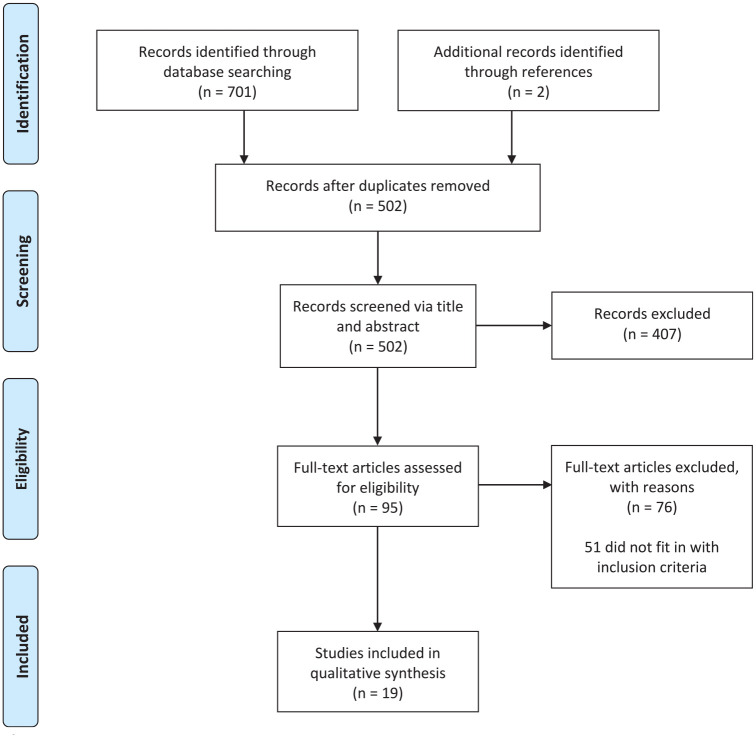
Search results.

The initial literature search identified 703 articles. After removal of duplicates, 502 studies remained. These underwent a 2-stage screening process performed independently by 2 reviewers. Primary screening involved reading titles and abstracts of the 502 articles, excluding 407 articles and leaving 95 for secondary screening. The full texts of the remaining articles were analyzed, and 19 studies^6,12-25^ were identified that fulfilled the criteria for inclusion in the qualitative analysis for the review (**Figure 3**). Of these articles, 5 were prospective studies,^13,16,21,22^ and 14 were retrospective studies (**Tables 2** and **3**).^6,12,14,15,17-20,23,25-29^ There was significant heterogeneity among study designs and recorded outcomes, meaning that a formal meta-analysis was not possible.

**Table 2.** Study Demographics.^a^

			Patients				Classification			ND	
Study	Type of study	Years of collection	Overall	With ND	Intervention	Primary tumor site	T	N	Stage	Level	UNI/BIL
Ghanem^12^	RET		4	4 (100)	TORS	Tonsil: 2 (50) BOT: 1 (25) Tonsil + BOT: (25)	T1: 1 (25) T2: 1 (25) T4: 2 (50)	N1: 2 (50) N2a: 1 (25) N2b: 1 (25)			
Rubek^13^	PRO	2014-2016	30	30 (100)	TORS	Tonsil: 21 (70) BOT: 7 (23) PPW: 2 (7)	T1: 14 (47) T2: 16 (53)	N0: 12 (40) N1: 10 (33) N2a: 1 (3) N2b: 7 (23)		II-IV: 30 (100)	UNI: 21 (70) BIL: 9 (30)
Cannon^14^	RET	2010-2016	88	88 (100)	TORS	Tonsil: 39 (44) BOT: 49 (56)	T1: 45 (51) T2: 34 (39) T3: 9 (10)	N0: 6 (7) N1: 13 (14) N2a: 15 (17) N2b: 48 (55) N2c: 3 (3) N3: 3 (3)	I: 2 (2) II: 4 (5) III: 13 (15) IVa: 66 (75) IVb: 3 (3)	II-IV: 88 (100)	UNI: 85 (97) BIL: 3 (3)
Kucur^15^	RET	2008-2013	113	113 (100)	TORS		T1: 43 (38) T2: 59 (52) T3: 8 (7) T4: 3 (3)	N0: 18 (16) N1: 11 (10) N2a: 33 (29) N2b: 38 (34) N2c: 8 (7) N3: 5 (4)	I: 7 (6) II: 4 (4) III: 12 (11) IVa: 81 (72) IVb: 9 (8)	I-V: 56 (50) I-IV: 25 (22) II-V: 8 (7) II-IV: 24 (21)	UNI: 97 (86) BIL: 16 (14)
van Loon^16^	PRO	2007-2012	18	9 (50)	TORS	Tonsil: 5 (56) BOT: 3 (33) Soft palate: 1 (11)	T1: 4 (44) T2: 5 (56)	N0: 9 (100)		I-IV: 9 (100)	UNI: 9 (100) BIL: 0 (0)
Granell^17^	RET		1	1 (100)	TORS	Tonsil: 1 (100)	T2: 1 (100)	N2b: 1 (100)			
Noel^18^	RET		1	1 (100)	TORS	Tonsil: 1 (100)	T2: 1 (100)	N1: 1 (100)	III: 1 (100)	II-IV: 1 (100)	
Olsen^19^	RET	2007-2009	18	18 (100)	TORS	Tonsil: 12 (67) BOT: 6 (33)	T1: 11 (61) T2: 6 (33) T3: 1 (6)	N0: 13 (72) N1: 2 (11) N2a: 1 (6) N2b: 2 (11)	I: 8 (44) II: 5 (28) III: 2 (11) IVa: 3 (17)		UNI: 17 (94) BIL: 1 (6)
Tsukahara^20^	RET		1	1 (100)	TORS	BOT: 1 (100)	T1: 1 (100)	N1: 1 (100)	I: (100)		
Genden^21^	PRO	April-Nov2007	20	11 (55)	TORS	Tonsil: 7 (64) BOT: 2 (18) Soft palate: 2 (18)	T1: 7 (64) T2: 4 (36)	N0: 6 (55) N1: 4 (36) N2: 1 (9)			UNI: 10 (91) BIL: 1 (9)
Krishnan^6^	RET	2008-2015	33	33 (100)	TORS		T1: 7 (21) T2: 19 (58) T3 3 (9) T4: 4 (12)	N0: 7 (21) N1: 1 (3) N2: 3 (9) N2a: 5 (15) N2b: 16 (48) N3: 1 (3)	II: 2 (6) III: 4 (12) IV: 27 (24)	I-V: 33 (100)	
Tsang^22^	PRO		1	1 (100)	TORS	Tonsil: 1 (100)	T1: 1 (100)	N1: 1 (100)		I-IV: 1 (100)	UNI: 1 (100)
Byeon^23^	RET	2011-2012	5	4 (80)	TORS	Tonsil: 4 (100)	T2: 3 (75) T3: 1 (25)	N2b: 4 (100)		II-V: 4 (100)	UNI: 2 (50) BIL: 2 (50)
Dabas^24^	PRO	2013-2015	57	57 (100)	TORS	Tonsil: 22 (39) BOT: 31 (54) Soft palate: 3 (5) PPW: 1 (2)	T1: 24 (42) T2: 33 (58)	N0: 49 (86) N1: 8 (14)	I: 19 (33) II: 30 (53) III: 8 (14)		UNI: 45 (79) BIL: 12 (21)
Parhar^25^	RET	2015-2019	20	20 (100)	TORS	Tonsil: 19 (95) BOT: 1 (5)	T1: 3 (15) T2: 6 (30) T3: 1 (5) T4: 10 (50)	N0: 9 (45) N1: 10 (50) N2: 1 (5)			
Jackel^26^	RET	2001-2005	6	5 (83)	TLM		T3: 4 (80) T4a: 1 (20)	N0: 2 (40) N2a: 2 (40) N3: 1 (20)			
Veit^27^	RET		1	1 (100)	TLM		T2: 1 (100)	N2c: 1 (100)		I-V: 1 (100)	UNI: 0 (0) BIL: 1 (100)
Leong^28^	RET		1	1 (100)	TLM	BOT: 1 (100)		N0: 1 (100)			UNI: 1 (100) BIL: 0 (0)
Moore^29^	RET	2007-2010	148	148 (100)	TORS						

Abbreviations: BIL, bilateral; BOT, base of tongue; ND, neck dissection; PRO, prospective; PPW, posterior pharyngeal wall; RET, retrospective; UNI, unilateral.

a Values are presented as No. (%). Blank cells indicate *not specified.*

**Table 3.** Primary Outcomes.^a^

Study	Patients with ND	Timing of ND^b^	Hemorrhage	Fistula formation	DDS/OS, %; mean follow-up	Recurrence rate
Ghanem^12^	4	Concurrent after	Major: 0 (0) Minor: 0 (0)	Intra: 0 (0) Post: 0 (0)		
Rubek^13^	30	Concurrent	Major: 1 (3) Minor: 2 (7)	Intra: 0 (0) Post: 0 (0)		
Cannon^14^	88	Concurrent		Intra: 2 (2) Post: 0 (0)	DSS: 95 OS: 100 2 y	2 (2)
Kucur^15^	113	Concurrent		Intra: 6 (5) Post: 0 (0)		
van Loon^16^	9	After (4 wk)	Major: 0 (0) Minor: 0 (0)	Intra: 1 (11) Post: 0 (0)	DSS: 89 OS: 100 2 y	1 (11)
Granell^17^	1	Before (2 wk)		Intra: 0 (0) Post: 0 (0)		
Noel^18^	1	Concurrent	Major: 0 (0) Minor: 0 (0)	Intra: 0 (0) Post: 0 (0)	DSS: 100 OS: 100 6 mo	0 (0)
Olsen^19^	18	Concurrent	Major: 0 (0) Minor: 0 0)	Intra: 0 (0) Post: 0 (0)	DSS: 78 OS: 94 2 y	4 (22)
Tsukahara^20^	1	Before (1 mo)	Major: 1 (100) Minor: 0 (0)	Intra: 0 (0) Post: 0 (0)	DSS: 100 OS: 100 1 y	0 (0)
Genden^21^	11	Concurrent	Major: 0 (0) Minor: 0 (0)	Intra: 1 (9) Post: 0 (0)	DSS: 100 OS: 100 4 mo	0 (0)
Krishnan^6^	33	Before: 8 (8 d) Concurrent: 19 After: 6 (10 d)	**Before** Major: 0 (0) Minor: 0 (0) **Concurrent** Major: 1 (3) Minor: 0 (0) **After** Major: 0 (0) Minor: 1 (3)	**Before** Intra: 0 (0) Post: 0 (0) **Concurrent** Intra: 3 (16) Post: 1 (5) **After** Intra: 2 (33) Post: 0 (0)		
Tsang^22^	1	Concurrent	Major: 0 () Minor: 0 (0)	Intra: 0 (0) Post: 0 (0)		
Byeon^23^	4	Concurrent before	Major: 0 (0) Minor: 0 (0)	Intra: 0 (0) Post: 0 (0)		
Dabas^24^	57	Concurrent before	Major: 1 (2)^c^ Minor: 0 (0)^c^		DSS: 88^c^ OS: 92^c^ 29 mo	2 (4)^c^
Parhar^25^	20	Concurrent after	Major: 0 (0) Minor: 0 (0)			
Jackel^26^	5	Concurrent before	Major: 0 (0) Minor: 1 (20)	Intra: 0 (0) Post: 0 (0)	DSS: 80 OS: 80 24.8 mo	1 (20)
Veit^27^	1	Concurrent			DSS: 100 OS: 100 12 mo	0 (0)
Leong^28^	1	Concurrent			DSS: 100 OS: 100 12 mo	0 (0)
Moore^29^	148	Concurrent		Intra: 42 (28) Post: 6 (4)		

Abbreviations: DDS, disease-specific survival; Intra, intraoperative; ND, neck dissection; OS, overall survival; Post, postoperative; TORS, transoral robotic surgery.

a Values are presented as No. (%) unless noted otherwise. Blank cells indicate *not specified.*

b Mean time between ND and TORS in parentheses.

c Eight patients with pathologically upstaged disease were excluded from these statistics.

Articles in this review were published between 2001 and 2020. The total number of patients who had TORS/TLM for primary tumor resection was 566. Of these, 546 also had an ND. The primary oropharyngeal sites were 54% tonsils, 42% base of tongue, 2% soft palate, 1% posterior pharyngeal wall, and 0.4% tonsil and base of tongue (n = 246). Five articles did not specify the primary cancer site.^6,15,26,27,29^

### Stage of Disease

The stage of disease was reported according to the seventh edition of the American Joint Committee on Cancer’s TNM classification. Tumor size (T) was cited in all but 2 studies,^28,29^ while nodal staging (N) was noted in all but 1 study.^29^ Across all studies, 41% of patients had T1 disease, 48% had T2 disease, 7% had T3 disease, and 5% had T4 disease (n = 397). Nodal disease was 33%, 16%, 48%, and 3% for N0, N1, N2, and N3 staged disease, respectively (n = 298). Overall cancer staging was reported in 7 studies and also showed large heterogeneity.^6,14,15,18-20,24^ The most common stage of disease was IV with 61% of patients being treated with this staging. A further 12% of patients were treated for stage I disease, while 14% were treated for stage II and 13% for stage III (n = 311).

### Neck Dissection

Two articles described ND as a separate procedure before TORS/TLM^17,20^; 3 as concurrent before procedures^23,24,26^; 10 as concurrent procedures^13-15,18,19,21,22,27-29^; 2 as concurrent after procedures^12,25^; 1 as a separate procedure after TORS/TLM^16^; and 1 as before, after, and concurrently to TORS/TLM.^6^

Of the 19 studies, 8 cited the level of ND.^6,13-16,18,22,23^ This accounted for 279 patients, of which 13% had I to IV, 32% had I to V, 51% had II to IV, and 4% had II to V. The ND was described in 11 studies as being unilateral or bilateral.^13-16,19,21-24,27,28^ Within these studies, 86% of patients had unilateral ND and 14% had bilateral ND (n = 333).

### Complications

Postoperative hemorrhage was divided broadly into major and minor bleeding. Major hemorrhage required surgical intervention (including arterial embolization) while minor bleeds recovered with conservative management. Of the 13 studies that recorded postoperative hemorrhage as an outcome, 4 cited major episodes of postoperative hemorrhage.^6,13,20,24^ In 2020, Tsukahara et al^20^ reported a patient having 2 episodes of severe pharyngeal bleeding, both requiring readmission. The second bleed led to hemorrhagic shock. There were 3 episodes of minor hemorrhagic bleeding across 2 studies.^13,26^

Altogether 15 studies with a total of 468 patients recorded fistula formation as a patient outcome.^6,12-23,26,29^ Of these, 12% had intraoperative fistulae, and 1% sustained postoperative fistulae. All intraoperative fistulae were managed in theater, with local flap reconstructions. However, in the study by Moore et al, 6 patients with intraoperative fistulae went on to develop postoperative fistulae.^29^

### Clavien-Dindo Classification Analysis

Of the 431 patients undergoing concurrent ND, 2 (0.5%) had grade III complications, 66 (15%) were classified as grade II, and 6 (1%) patients had grade V complications. Of the 39 patients with ND performed after transoral surgery (including the concurrent after and after cohorts), 3 (8%) had grade II complications and 1 (3%) had grade V. Seventy-six patients had ND prior to transoral surgery (including the before and concurrent before cohorts). Of these, 1 patient (1%) had grade IV complications, 2 (3%) were grade II, and 6 (8%) had grade V.

### Disease-Specific Survival and Overall Survival

Ten studies described DSS and OS, with varying follow-up times. Five studies cited DSS and OS as 100% for 13 patients at follow-up times ranging from 2 months to 1 year.^18,20,21,27,28^ Three studies with a 2-year follow-up period found DSS to be 95%, 89%, and 78% while OS was at 100%, 100%, and 94%.^14,16,19^ Dabas et al^24^ cited a DSS of 88% and OS of 92% at a mean follow-up time of 29 months, and Jackel^26^ reported DSS and OS at 80% with a mean follow-up of 24.8 months. Ten studies (192 patients) recorded a recurrence rate, which was 5% on average.^14,16,18-21,24,26-28^ Five studies described no recurrence.^18,20,21,27,28^

### Effect of ND Timing

Across the studies, 12% had concurrent before procedures; 4% had concurrent after procedures; 2% had ND as a separate procedure before (minimum 8 days and maximum 1 month before TORS/TLM); 3% had ND as a separate procedure after (minimum 10 days and maximum 8 weeks after TORS/TLM); and 79% patients had a concurrent procedure. The timing of ND was not mentioned in this cohort (n = 546).

In patients with ND before TORS/TLM (including concurrent before and before cohorts), 3% experienced major bleeding and 1% experienced minor bleeding, while fistula rates were at 0% (n = 76). Of patients with ND after TORS/TLM (including concurrent after and after cohorts), 3% experienced minor hemorrhage, and 8% had intraoperative fistulae (n = 39). In the concurrent cohort of patients, 1% experienced major bleeds and 0.3% had minor bleeds. A further 13% developed intraoperative fistulae and 2% developed postoperative fistulae (n = 431). Recurrence rates were 4% in patients who had ND before TORS/TLM and 11% in patients who had ND after TORS/TLM. In the cohort of concurrent ND and TORS/TLM, the recurrence rate was 1%.

### Bias Assessment

The articles in this review were predominantly nonrandomized studies and were reviewed for bias with the ROBINS-I tool (**Table 4**),^10^ with case reports classified as “severe” bias.^17,18,20,22,27,28^ Selection bias in disease severity and stage, different inclusion and exclusion criteria, lack of common outcome measures and varying lengths of follow-up were identified as some of the factors increasing the bias levels in the articles.

**Table 4.** Bias Assessment of Studies With the ROBINS-I Tool.^a^

Study	D1	D2	D3	D4	D5	D6	D7	Overall bias
Ghanem^12^	Low	Low	Low	Low	Low	Low	Low	Low
Rubek^13^	Low	Low	Low	Moderate	Low	Low	Low	Moderate
Cannon^14^	Low	Low	Low	Moderate	Low	Low	Low	Moderate
Kucur^15^	Low	Low	Low	Low	Low	Low	Low	Low
van Loon^16^	Low	Moderate	Low	Moderate	Low	Low	Low	Moderate
Granell^17^	Low	Serious	Serious	Low	No information	Moderate	Low	Serious
Noel^18^	Low	Serious	Serious	Moderate	Low	No information	No information	Serious
Olsen^19^	Low	Low	Low	Low	Low	Low	Low	Low
Tsukahara^20^	Low	Serious	No information	Low	Low	Low	Low	Serious
Genden^21^	Low	Low	Low	Low	Low	Low	Low	Low
Krishnan^6^	Low	Low	Low	Low	Low	Low	Low	Low
Tsang^22^	Low	Serious	Serious	Low	Low	Moderate	Low	Serious
Byeon^23^	Low	Low	Low	Low	Low	Low	Low	Low
Dabas^24^	Low	Low	Low	Moderate	Moderate	Low	Low	Moderate
Parhar^25^	Low	Low	Low	Moderate	Low	Low	Low	Moderate
Jackel^26^	Moderate	Low	Low	Moderate	Low	Low	Low	Moderate
Veit^27^	Low	Serious	Serious	Low	Moderate	Low	Low	Serious
Leong^28^	Low	Serious	Serious	Low	Low	Low	Low	Serious
Moore^29^	Low	Low	Low	Low	Low	Low	Low	Low

a D1, bias due to confounding; D2, bias in selection of participants into the study; D3, bias in classification of intervention; D4, bias due to deviations from intended interventions; D5, bias due to missing data; D6, bias in measurement of outcomes; D7, bias in selection of reported result.

## Discussion

When considering management of patients presenting with oropharyngeal cancer, there is a divergence in approaches; some have been identified in this review. Generally, ND timing lacks standardization and varies among centers. This review focuses on transoral surgery in conjunction with ND and aims to shed light on whether the timing of ND has any effect on patient outcomes.

Repanos et al in 2017 published a similar systematic review looking at the timing of ND in relation to transoral surgery, including TORS and TLM.^30^ The review included articles that failed to mention ND timing, as well as articles in which not all patients had ND. Case reports were also excluded from the review. The modalities analyzed were transoral laser surgery and TORS for resection of head and neck squamous cell carcinoma. The results indicated that timing of ND did not affect OS and highlighted the lack of robust evidence in the literature regarding patient complications and oncologic outcomes with respect to timing of ND in conjunction with primary surgery.

To date there have been no randomized controlled trials (RCTs) assessing timing of ND in conjunction with transoral surgery, although prospective and retrospective studies on this topic have been performed.^8,31^ Frenkel et al^31^ retrospectively analyzed 386 procedures in New York State. Patients had ND, performed concurrently, before, and after TORS. Patient outcomes were not recorded in this study as it predominantly focused on the economic implications of ND timing. The study gathered objective data, showing that concurrent ND with TORS is cost-effective as it maximizes usage of expensive medical equipment and reduces patients’ length of stay. It was found that the difference in mean prices for staged procedures as compared with concurrent procedures was >$30,000.

### Hemorrhage and Fistula Rates

There were insufficient data from the studies in this review to draw meaningful conclusions about whether the timing of ND affects postoperative hemorrhage rates. Six studies (352 patients) did not identify hemorrhage as an outcome.^14,15,17,27-29^ Of the remaining patients who had concurrent ND, 3% had major bleeds and 3% had minor bleeds recorded,^6,13^ with an overall bleeding rate of 5% (n = 80). In both instances of major bleeding, vessel ligation was not performed during the initial procedure. In patients with ND performed after TORS/TLM, 3% experienced minor bleeding (n = 39).^6^ In patients with ND performed before TORS/TLM, 3% had a major bleed,^20,24^ and 1% had a minor bleed (n = 75).^26^ In the case report by Tsukahara et al,^20^ external carotid artery ligation did not occur until the patient was readmitted for the second episode of pharyngeal bleeding. In the majority of patients with major bleeding, vessel ligation did not occur regardless of ND timing. In all 4 episodes of major hemorrhage, bleeding was stopped with readmission and vessel ligation.^6,13,20,24^

Four studies (79 patients) did not include fistula rates as outcome measures.^24,25,27,28^ Of the concurrent cohort of patients, 13% had intraoperative fistulae, and 2% had postoperative fistulae (n = 429). There were no recorded fistulas in patients with ND before TORS/TLM (n = 19). In patients with ND after TORS/TLM (including concurrent after), 16% reported intraoperative fistulae^6,16^ and no postoperative fistulae were noted (n = 19). Due to the variability in the sample size of each cohort, definitive conclusions cannot be made about fistula formation. However, the trends identified in this study (increased fistula rate in patients with concurrent ND) are in keeping with published literature.

Moore et al^29^ found that 29% of patients developed intraoperative communications and that 4% resulted in delayed fistula formation in patients undergoing concurrent transoral surgery and ND (n = 148). Their results showed that fistulae occur regardless of T stage but generally correlate with advanced-stage neck disease, suggesting that there is an increased probability of fistulae formation when treating stage III and IV oropharyngeal disease.

### Overall Survival and Disease-Specific Survival

Long-term patient outcomes such as DSS and OS were not mentioned in 9 studies.^6,12,13,15,17,22,23,25,29^ In studies reporting DSS and OS for concurrent ND, the mean DSS was calculated to be 96% and mean OS was 99%, with a follow-up period ranging from 2 to 29 months.^15,18,19,21,27,28^ One study cited DSS and OS rates of 89% and 100% at 2 years, respectively, for patients undergoing ND after TORS/TLM.^16^ Three studies described mean DSS and OS rates of 89% and 91% at a follow-up ranging from 1 year to 29 months in 58 patients undergoing ND before (including concurrent before) TORS/TLM.^20,24,26^ It is important to note that while we have reported DSS and OS, interpretation of these data should be cautious due to the lack of TNM-stratified survival rates within the studies in this review. The number of studies reporting DSS and OS as outcomes for each category of ND (before, concurrent, and after) was too small to draw definitive conclusions.

### Level of ND

Besides ND timing, one variation identified among studies in this review is the level of ND performed. Most authors recommended a selective ND of levels II to IV for OPSCC treatment.^18^ Performing level I ND in patients with OPSCC carries added risk of creating PCF intra- or postoperatively. Moreover, the rate of occult level I metastases based on preoperative evaluation is estimated to be 3%,^32^ which is below the threshold to indicate standard inclusion of this level according to standard UK practice. The current guideline for surgical management of these patients in the United Kingdom is that ND should include levels II to IV and possibly level I.^33^ This is reflected in the results of our study, with 100% of patients having ND of levels II to IV and 64% having ND of level I, while 36% had level V. In 2003, Doweck et al^34^ performed a study of 76 patients, looking at the extent of ND required in oropharyngeal cancer. They concluded that surgical management of oropharyngeal cancer should include a selective ND of levels II to IV and that without radiologic and clinical evidence of positive nodes in level I and V, these levels could be spared.

### Limitations

A major issue encountered when performing this review was interpreting the findings of the studies. The literature search did not identify any RCTs, which limited analysis. In addition to the 19 articles in the review, only 5 were prospective studies. Therefore, the articles reviewed showed variation in design and outcome measures, and the lack of control arms in the studies added to the heterogeneity among the articles. This limited statistical analysis as a meta-analysis could not be performed. Individual patient-level analysis was not possible to extract from many of the studies.

### Potential for Bias

We declare no biases in the construction of this review. A thorough search was conducted by 2 independent reviewers; the search was limited to the English language. Articles studying cancers outside the oropharynx (including the oral cavity), modalities other than TORS/TLM, and those failing to distinguish ND timing as a feature in the results were excluded.

### Future Implications

The evidence presented in this review is insufficient to draw definitive conclusions surrounding ND timing and patient outcomes. As such, practice should continue to reflect the decision-making process of the multidisciplinary team. More research should be conducted, including RCTs, to allow for a more thorough review to be completed before any conclusive decisions arise regarding ND timing. In addition, other factors should be considered when looking at ND timing, including cost-effectiveness of performing staged ND, the level of ND, anaesthetic risk to the patient with having 2 procedures, and the effects of potentially having delayed adjuvant treatment.

## Conclusion

In conclusion, transoral surgery, TORS in particular, has become a well-established modality for treating oropharyngeal carcinoma. Given the increasing rates of these cancers, the role of TORS/TLM is becoming more relevant.

This review demonstrates the lack of robust literature when analyzing ND timing in relation to TORS/TLM for oropharyngeal carcinoma. There should be a focus on producing more evidence for patient outcomes surrounding TORS/TLM with concurrent or staged ND. Wherever possible, this evidence should be in the form of RCTs or prospective studies, although it is acknowledged that these would raise ethical concerns regarding patient allocation to particular treatment arms prospectively.

Due to the heterogeneity of existing studies and the lack of comparator arms, meta-analysis could not be performed. Pooled analysis was conducted for certain outcomes, where this was possible. There are insufficient data to comment on whether the timing of ND in relation to TORS affects the outcomes of patients. However, within the limitations of the current evidence base, there seems to be no correlation between timing of ND and complications.

Finally, heterogeneity was identified in the extent of ND routinely performed for oropharyngeal carcinoma. Therefore, a dedicated systematic review on this topic would likely be beneficial in providing the best possible quality evidence for clinicians in assessing the necessity of level I ND in patients with oropharyngeal cancer.

## Author Contributions

**Jai Parkash Ramchandani**, wrote manuscript, involved in study design, data acquisition and analysis, drafting, final review of the manuscript prior to submission, study supervision; **Aina Brunet-Garcia**, wrote manuscript, involved in study design, data acquisition and analysis, drafting, final review of the manuscript prior to submission; **Nikoleta Skalidi**, involved in study design, data acquisition and analysis, critical review of manuscript and final review prior to submission; **Jack Faulkner**, involved in critical review of manuscript and final review prior to submission; **Aleix Rovira**, involved in critical review of manuscript and final review prior to submission; **Ricard Simo**, involved in critical review of manuscript and final review prior to submission; **Jean-Pierre Jeannon**, involved in critical review of manuscript and final review prior to submission; **Asit Arora**, involved in critical review of manuscript and final review prior to submission.

## Disclosures

**Competing interests:** None.

**Sponsorships:** None.

**Funding source:** None.
